# Prevalence of Gestational Toxoplasmosis by Race and Ethnicity: A Systematic Review

**DOI:** 10.3389/phrs.2026.1609202

**Published:** 2026-04-07

**Authors:** Sara Rafaela Valcacio Camargo, Raquel Souza Borges, Samantha Valcacio Camargo, Ana Karoline de Freitas Nascimento, Héllyda de Souza Bezerra, Talita Araujo Souza, Dyego Leandro Bezerra de Souza, Isabelle Ribeiro Barbosa

**Affiliations:** 1 Federal University of Rio Grande do Norte, Natal, Brazil; 2 Universidade Federal de Pernambuco, Recife, Brazil

**Keywords:** ethnicity, pregnant women, prevalence, race, toxoplasmosis

## Abstract

**Objectives:**

to identify the prevalence of toxoplasmosis during pregnancy according to race or ethnicity through a systematic review.

**Methods:**

the protocol was registered in PROSPERO (CRD42024596261). Searches were conducted in PubMed, LILACS, Web of Science, Scopus, CINAHL, and Google Scholar using descriptors related to toxoplasmosis, pregnancy, prenatal care, and race/ethnicity combined with Boolean operators. Cross-sectional studies reporting prevalence among pregnant women according to racial or ethnic groups were included.

**Results:**

of the 4,652 records identified, 10 studies met the inclusion criteria. Data were extracted, organized, and analyzed qualitatively. The findings revealed differences in toxoplasmosis prevalence between ethno-racial groups. In Brazil, higher prevalence was observed among Pardo (mixed-race) and Indigenous populations. In the United Kingdom, Afro-Caribbean women showed a prevalence of 31.48%. In Sri Lanka, Sinhalese women had higher prevalence (13.56%) compared to non-Sinhalese women (6.3%). Variations across continents were evident.

**Conclusion:**

significant disparities in toxoplasmosis prevalence during pregnancy were identified among ethno-racial groups, especially among Pardo, Indigenous, and Black populations, highlighting the need for broader multicenter investigations worldwide.

**Systematic Review Registration:**

https://www.crd.york.ac.uk/prospero/display_record.php?RecordID=CRD42024596261, identifier CRD42024596261.

## Introduction

Toxoplasmosis is an infectious disease caused by the protozoan *Toxoplasma gondii*, which affects both humans and animals [1]. It is considered a highly widespread zoonosis with a wide geographical distribution, regarded as one of the most common in humans [2]. The main forms of transmission of this protozoan include food, direct contact with contaminated material, and vertical transmission [3].

Transplacental infection by *T. gondii* is called congenital toxoplasmosis [4] and can result in miscarriage, prematurity, infant morbidity and mortality, and neurological and ophthalmic impairments [5, 6]. Prevention, based on health education and prenatal serological screening [7], is essential to reduce adverse maternal and infant outcomes [8].

Toxoplasmosis has a high worldwide prevalence among pregnant women, estimated at 32.9% [9]. However, its distribution is not uniform, with significant differences between regions: Americas (45.2%), Eastern Mediterranean (39.7%), Africa (36.5%), Europe (30.0%), Southeast Asia (24.6%), and Western Pacific (11.2%) [10]. These data highlight the widespread global dissemination of the infection and emphasize its relevance as a public health problem in different contexts.

Infection by the protozoan is influenced by several cultural, social, and environmental factors, which play a predictive role in exposure to *T. gondii*. Aspects such as income, social and physical environment, education, race/ethnicity, and access to basic sanitation are directly related to vulnerability to infection [11].

Within the scope of social markers of disparity, race and ethnicity constitute central categories for understanding health inequities. Although frequently used interchangeably in scientific discourse, they represent distinct constructs. Race is contemporaneously understood as a social construction historically associated with processes of racialization, which classify individuals based on phenotypic characteristics such as skin color and physical traits. In contrast, ethnicity refers to the identification of human groups based on shared sociocultural elements, including ancestry, language, territory, and historical traditions [12, 13].

The inequities faced by women from some ethnic-racial groups reflect structural inequalities deeply rooted in historical, social, and economic factors. Black, indigenous, and other women belonging to ethnic-racial minorities often face greater vulnerability due to a combination of racial discrimination, gender inequality, and socioeconomic precariousness, which makes them more vulnerable to risk factors and barriers to access to screening, early diagnosis of the disease, and quality care [14].

A study by Leal et al. [15] showed that black women have higher maternal mortality rates than non-black women, in addition to experiencing prenatal care with fewer consultations and exams, less access to information, and a higher frequency of neonatal problems in their children [15]. Similarly, a systematic review pointed out that black women had the lowest prevalence of access to prenatal services in the first trimester when compared to white women and women from other racial groups [16].

These findings are consistent with the study by Van Daalen et al. [17], which suggests a positive association between racial discrimination and worse pregnancy outcomes, such as premature birth and small for gestational age newborns. Among Hispanics, Indigenous people, and mestizos, those perceived socially as white had significantly better health than those perceived as non-white [17].

Thus, it is essential to investigate whether race or skin color has a direct impact on the prevalence of toxoplasmosis in pregnancy, as this may reveal racial inequalities with severe and lasting consequences. This debate can also inform the formulation of public policies aimed at racial equity in maternal care, supported by a robust technical and scientific basis to improve the quality of care in obstetrics and perinatal medicine.

The objective of this study was to identify the prevalence of toxoplasmosis during pregnancy compared by ethnic and racial aspects through a systematic review.

## Methods

### Protocol and Registration

To conduct this systematic review, the protocol was developed and registered in the International Prospective Register of Systematic Reviews (PROSPERO) under the number CRD42024596261. The PRISMA (Preferred Reporting Items for Systematic Reviews and Meta-Analysis) guidelines [18] were used as a writing guide. In this study, the research question focused on: Is there a difference between ethnic-racial groups in the prevalence of toxoplasmosis in pregnant women?

### Eligibility Criteria

The inclusion and exclusion criteria for articles obtained during database searches followed the PECO strategy – Population, Exposure, Comparison, and Outcome, in which the Population is pregnant women, including adolescents, without restriction of nationality and at any gestational age; Exposure – infection with Toxoplasma Gondii during pregnancy; Comparison – pregnant women of different races and ethnicities; Outcome – Prevalence of toxoplasmosis during pregnancy in black, white, and other ethnic women—type of studies – Cross-sectional studies. Cohort studies, case-control studies, and literature reviews (integrative, narrative, or systematic with or without meta-analysis), case series, case reports, and qualitative analyses were not included in this research.

Given the scarcity of studies specifically designed to investigate racial inequalities in gestational toxoplasmosis, we chose to include investigations that reported the race/ethnicity variable in the description of infection prevalence, regardless of whether adjusted analyses were performed. This methodological decision was grounded in the objective of the present review, which was to map and synthesize the current state of available evidence regarding the distribution of toxoplasmosis during pregnancy across ethno-racial groups. Consequently, both studies featuring adjusted analyses and those with exclusively descriptive presentations were considered eligible, allowing for the identification of methodological gaps, heterogeneities in variable operationalization, and analytical limitations within the existing literature.

### Search Strategy and Data Sources

In September 2024, the following electronic databases were searched: PubMed, Web of Science, LILACS, Scopus, CINAHL, and Google Scholar to identify articles in the gray literature, in addition to manual searches. The searches were conducted without restrictions on the language of the articles or date of publication.

A librarian specializing in the field was consulted to develop the search strategies. To capture a wide range of articles related to the topic, the following health sciences descriptors (DeCS/MeSH) were used “Toxoplasmosis,” “Toxoplasma infection,” “Toxoplasma gondii,” “Pregnant women,” “Pregnancy,” and “Prenatal Care”, in addition to related free terms, combined with the Boolean operators “AND” and “OR” to obtain the most appropriate search key in each database, according to the controlled vocabulary. The search strategies used in this study are presented in Table 1.

**TABLE 1:** Search strategy (Brazil, 2025).

Database	Strategy
Pubmed	(Toxoplasmosis OR toxoplasma infection OR toxoplasma gondii OR T. gondii OR toxoplasma) AND (pregnant women OR pregnancy OR gestation OR prenatal care OR antenatal care)
Web of science	(Toxoplasmosis OR toxoplasma infection OR toxoplasma gondii OR T. gondii OR toxoplasma) AND (pregnant women OR pregnancy OR gestation OR prenatal care OR antenatal care)
Lilacs	(“Toxoplasma gondii” OR “toxoplasmosis”) AND (“woman” OR “pregnant woman” OR “pregnancy” OR “prenatal”)
Scopus	(Toxoplasmosis OR toxoplasma infection OR toxoplasma gondii OR T. gondii OR toxoplasma) AND (pregnant women OR pregnancy OR gestation OR prenatal care OR antenatal care)
Cinahl	(Toxoplasmosis OR toxoplasma infection OR toxoplasma gondii OR T. gondii OR toxoplasma) AND (pregnant women OR pregnancy OR gestation OR prenatal care OR antenatal care)
Google scholar	(Toxoplasmosis OR toxoplasma infection OR toxoplasma gondii OR T. gondii OR toxoplasma) AND (pregnant women OR pregnancy OR gestation OR prenatal care OR antenatal care)

Initially, exploratory search tests were conducted including terms related to race or skin color. However, the mandatory inclusion of these descriptors substantially reduced the sensitivity of the search strategy. In some tests, the strategy that combined terms for toxoplasmosis in pregnancy with race/ethnicity descriptors resulted in very limited retrieval of records and failed to identify articles that were previously known and considered relevant to the topic.

This result may be explained by the well-recognized terminological heterogeneity and the inconsistent indexing of the race/ethnicity variable in bibliographic databases. In many epidemiological studies, race or ethnicity is not the primary exposure variable and is often reported only in the sample characterization or included as a covariate in multivariable analyses. In such situations, these variables do not always appear in titles, abstracts, or indexed descriptors, which reduces the probability of retrieval when race/ethnicity-related terms are required in the search strategy.

Indeed, it was observed that key studies on toxoplasmosis in pregnancy that report information on race or ethnicity were not retrieved when these descriptors were included in the search strategy, but were identified when the search was conducted using only terms related to gestational toxoplasmosis. This finding suggests that requiring these descriptors could lead to the inadvertent exclusion of potentially relevant studies.

Therefore, a more sensitive search strategy was adopted, centered on terms related to toxoplasmosis during pregnancy. The identification of the race/ethnicity variable and its analytical role in the included studies was subsequently performed during the screening and full-text review stages, according to the criteria previously defined in the registered protocol.

### Screening and Selection of Studies

The results obtained from each database were exported to the Mendeley Desktop® reference manager, version 2.85.0, to check and eliminate duplicate articles. Subsequently, they were deposited in the Rayyan QCRI® software for blind reading of the articles, which was carried out in two stages: in the first stage, screening was performed according to the reading of titles and abstracts, and subsequently, in the second stage, the articles were read in their entirety. In both stages, eligibility criteria were considered, and the articles were read by two independent reviewers (SRVC and RSB) trained in study selection, use of analysis software, and data extraction. Conflicts were resolved in consensus meetings by a third reviewer (IRB).

### Risk of Bias Assessment

To assess the methodological quality of each included study, the Joanna Briggs Institute’s critical appraisal tool for systematic reviews of cross-sectional studies was used. This tool consists of eight questions that assess the methodological quality of the research and determine the extent to which the study addresses the possibility of bias in its design, conduct, and analysis [29].

The questions were classified independently by the authors as “Yes,” “No,” “Unclear,” or “Not/Applicable.” The classification of the risk of bias results will be evaluated as: (1) low risk, if the studies achieved more than 70% “yes” scores; (2) moderate risk, if the “yes” score is between 50% and 69%; and (3) high risk of bias, if the bias score is less than 49% [30]. Review Manager software (RevMan; version 5.4.1) was used to prepare the figure.

### Data Extraction

The authors extracted the data using Microsoft Excel. The data extracted from the included articles were authors, year of publication, study location, sample size, age group of the population, race/ethnicity of the population, diagnostic test method, Sero reaction, prevalence of toxoplasmosis, measures of association (odds ratio), and adjustment variables.

Given the lack of international standardization in the definition and operationalization of race and ethnicity, as well as the conceptual heterogeneity across different sociocultural contexts, these variables were analyzed in this review as reported in the included primary studies. For analytical purposes, race and ethnicity were considered collectively as sociodemographic markers of population stratification, maintaining the original terminology adopted by the authors. No reclassification or terminological harmonization was performed between studies to preserve methodological fidelity and avoid artificial interpretations resulting from recategorization.

## Results

### Study Selection and Characteristics

**FIGURE 1:**
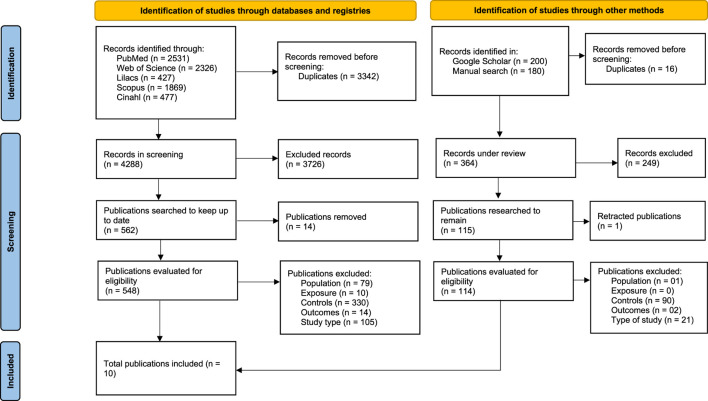
Flowchart of the study search and selection process (Brazil, 2025).

Searches of databases and gray literature resulted in 4,652 studies. After removing duplicate articles, the studies were screened by title and abstract, resulting in 677 articles potentially eligible for full-text reading. Of these, 15 were excluded because the full text was unavailable. At the end of the selection process, 10 studies met the eligibility criteria and were included in this systematic review (Figure 1).

### Synthesized Findings

Table 2 summarizes the characteristics of the included studies. Most of the articles included (09) originate from the American continent, including Brazil and Mexico, as well as the Asian continent. There was only one study from Europe, conducted in the United Kingdom [25]. Brazil stands out with several regions represented, with studies conducted in the cities of Ilhéus-Bahia, Foz do Iguaçu-Paraná, in the state of Goiás, and in the city of Porto Alegre, in Rio Grande do Sul. The age ranges of the participants varied widely, ranging from adolescents under 18 to women over 50, as in studies conducted in Brazil and the United Kingdom. Other studies, such as that by Chandrasena et al. [19] in Sri Lanka, included women aged 16 to 40 [19], while in Aguascalientes, Mexico [20], the age range was 13–42. This age range reflects the search for a broad analysis, covering different reproductive profiles and their associated risks for toxoplasmosis.

**TABLE 2:** Characteristics of included studies (Brazil, 2025).

Author (year)	Location of Study	Age group	Examination method	Sample size	Race/ethnicity of the population	Prevalence of toxoplasmosis	Seroreaction	Odds ratio (95% CI)	Adjustment variables
Chadrasena et al. [19]	Western province of Sri Lanka	16–40	LFA	244 48	Sinhala Non-Sinhala	13.56 6.3	IgG	1 0.352 (0.99–1.25)	Age group educational level Occupation Marital status
Alvarado-esquivel et al. [20]	Aguascalientes Mexico	13–42	ELISA	312 4	Mixed race White	5.4 75.0	IgG	1 149.4 (10.8–2054.1)	Educational level Frequency of eating out washing hands before eating Sanitary facilities
Costa et al. [21]	Ilheus Brazil	13–44	ELISA	61 116 271 2 6	White Black Brown Yellow Native	54.09 28.44 74.54 100 83.33	IgG	White vs. Non-white 2.26 (1.25–4.06)	Income Pregnancy history
Sartori et al. [22]	Goiás Brazil	<19 ->31	ELISA	3,046 4,813 570	White Brown Black	64.2 69.1 72.1	IgG	1 1.4 (1.1–1.6) 1.6 (1.1–1.7)	Age group Education
Varella et al. [23]	Porto Alegre Brazil	13–45	MEIA	948 67 246	White Mixed Black	58.9 61.1 63.0	IgG and/or IgM	1 0.99 (0.64–1.54) 1.11 (0.87–1.43)	Age group Education Origin
Ramsewak et al. [24]	Trinidad and Tobago	<20–50	EIA	106 38 86 2	African East Indian Mestizo Other	31.1 42.1 44.2 50.0	IgG or IgM	Not present	-
Flatt and Shetty^a^ [25]	London United Kingdom	16–49	ELISA	2013 162 166 76 43 150	White Caucasian Afro-Caribbean Indian subcontinent Far East Middle East Mixed	15.75 31.48 14.46 13.16 34.88 23.33	IgG	1 2.67 (1.83–3.88) 0.98 (0.62–1.57) 0.82 (0.41–1.62) 3.12 (1.62–5.99) 1.75 (1.16–2.63)	Eating undercooked meat Drinking unpasteurized milk Eating unpasteurized cheese Cat breeder
Enter et al. [26]	Thailand and Myanmar	16–46	ELISA	121 57	Karen Burmese	28.92 35.08	IgG and IgM	Not present	-
Chemoh et al. [27]	Malaysia	20 ->40	ELISA	161 28 27	Malaysian Chinese Indian	40.4 17.9 22.2	IgG and/or IgM	Not present	-
Mohamed et al. [28]	Makkah Al Mukarramah, Saudi Arabia	16–40	ELISA	206 19 12	Arabic Asian African	23.78 26.31 9.09	IgG	Not present	-

^a^ In this study, race/ethnicity was analyzed as the primary exposure variable. In the remaining studies, race/ethnicity was included only as a covariate or descriptive sociodemographic variable.

The analysis of the studies included the detection of IgG, IgM, or both antibodies, depending on the objectives of each study. Most studies used IgG Sero reaction, which indicates past infections and is a valuable marker for understanding the cumulative prevalence of toxoplasmosis over time. In some cases, such as in the study conducted in Porto Alegre [23], both IgG and IgM were evaluated, allowing the identification of recent infections, which are especially relevant in contexts of gestational risk. Studies that analyzed IgM often complemented the results with avidity tests, ensuring the differentiation between recent and old infections.

The studies included in the analysis used different serological methods to detect the presence of antibodies against *T. gondii*, with emphasis on the ELISA (Enzyme-Linked Immunosorbent Assay) test, used in most studies due to its high sensitivity and specificity for IgG and IgM. Other methods used were MEIA (Microelisa Automated Immunoassay), LFA (Lateral Flow Assay), and EIA (Enzyme Immunoassay). These tests allowed the identification of previous, recent, or acute infections, depending on the detection of IgG, IgM, or both antibodies. In some cases, avidity tests were performed to distinguish acute infections from past infections. The choice of method varied according to the resources available, the context of the study, and the research objectives.

High heterogeneity was observed in the operationalization of the race and ethnicity variables across the ten included studies. Chandrasena et al. and Alvarado-Esquivel et al. [19, 20] adopted dichotomous classifications, such as “White vs. Non-white,” or nationality-based categories obtained through questionnaires and self-reporting [19, 20]. In contrast, Costa et al, Sartori et al. and Varella et al. [21–23] utilized polytomous (multicategorical) categories following the Brazilian Institute of Geography and Statistics (IBGE) criteria, also based on questionnaires and self-declaration [21–23].

Ramsewak et al., Flatt and Shetty, Chemoh et al., Mohamed et al. and Enter et al. [24–28] employed multicategorical categories grounded in nationality, geographic region of origin, or ethnic-based groups [24–28]. These variables were used as proxy markers for ethnicity, lacking a clear conceptual definition regarding the distinction between race, ethnicity, and nationality.

There is a difference between the racial groups studied, with the prevalence of toxoplasmosis varying widely among populations of different ethnic and racial origins. In studies conducted in Brazil, such as in Ilhéus, in the state of Bahia [21], the prevalence was higher among brown-skinned individuals (74.54%) and indigenous individuals (83.33%). The prevalence of toxoplasmosis among black pregnant women in a study conducted in Goiás-GO was 72.1% [22], while in Porto Alegre-RS it was 63.0% [23].

In Trinidad and Tobago, the prevalence of toxoplasmosis was 31.1% for African pregnant women [24], and in the United Kingdom, the prevalence was 31.48% among Afro-Caribbean women [25], highlighting differences between continents. On the other hand, in Sri Lanka, the prevalence was higher in Sinhalese women (13.56%) compared to non-Sinhalese women (6.3%) [19].

Beyond the prevalence distribution across ethno-racial groups, several studies conducted multivariable analyses to investigate factors associated with toxoplasmosis seropositivity. Statistically significant associations between race/ethnicity and infection were observed in different contexts. In Ilheus [21], non-white women showed a higher likelihood of seropositivity compared to white women (OR = 2.26; 95% CI: 1.25–4.06). In Goiás [22], Pardo (OR = 1.4; 95% CI: 1.1–1.6) and Black women (OR = 1.6; 95% CI: 1.1–1.7) had a greater probability of infection relative to white women. In the United Kingdom [25], Afro-Caribbean pregnant women exhibited higher odds of seropositivity (OR = 2.67; 95% CI: 1.83–3.88), even after adjusting for dietary habits. Furthermore, factors such as age, education level, income, occupation, and dietary behaviors were also associated with the infection across various adjusted models.

These data highlight significant variation between regions, suggesting that factors such as socioeconomic conditions, environmental exposure, and cultural differences may influence the observed rates. In addition, due to methodological and population heterogeneity, it was not possible to perform the meta-analysis planned in the protocol.

### Risk of Bias Assessment

**FIGURE 2:**
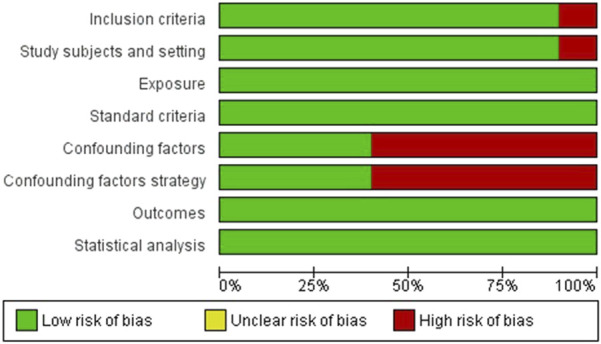
Bias risk assessment chart (Brazil, 2025).

Regarding the risk of bias, most studies were classified as low risk [20–23, 25–28] and only two as moderate risk [19, 24]. Among the individual items, all studies measured exposure validly and reliably, used objective standards and criteria to measure the condition, measured outcomes with valid and reliable methods, and performed appropriate statistical analyses. However, more than half of the studies did not identify confounding factors [19, 22, 25–28]. Among these studies, only four report strategies for dealing with confounding factors [21–23, 25]. These aspects are shown in Table 3; Figure 2.

**TABLE 3:** Methodological quality and risk of bias analysis according to the Joanna Briggs critical appraisal checklist for analytical cross-sectional studies (Brazil, 2025).

1	2	3	4	5	6	7	8	9	10	Item
-	+	+	+	+	+	+	+	+	+	Were the criteria for inclusion in the sample clearly defined?
+	+	+	+	+	-	+	+	+	+	Were the study subjects and the setting described in detail?
+	+	+	+	+	+	+	+	+	+	Was the exposure measured in a valid and reliable way?
+	+	+	+	+	+	+	+	+	+	Were objective, standard criteria used for measurement of the condition?
-	-	+	+	+	-	+	-	-	-	Were confounding factors identified?
-	-	+	+	+	-	+	-	-	-	Were strategies to deal with confounding factors stated?
+	+	+	+	+	+	+	+	+	+	Were the outcomes measured in a valid and reliable way?
+	+	+	+	+	+	+	+	+	+	Was appropriate statistical analysis used?
MR	LR	LR	LR	LR	MR	LR	LR	LR	LR	Evaluation

1.Chadrasena et al [19]; 2. Alvarado-Esquivel et al [20]; 3. Costa et al [21]; 4. Sartori et al [22]; 5. Varella et al [23]; 6. Ramsewak et al [24]; 7. Flatt and Shetty [25]; 8. Enter et al [26]; 9. Chemoh et al [27]; 10. Mohamed et al [28].

“+” = Yes; “-” = no.

LR: Low Risk of Bias MR: Moderate Risk of Bias HR: high risk of bias.

## Discussion

The systematic review provided data on the prevalence of toxoplasmosis in pregnant women ranging from 5.4% to 83.33%, associated with race, ethnicity, educational level, age group, income, occupation, eating habits, and hand washing before eating. The included studies used race and ethnicity as synonyms and, in some cases, employed nationality or geographic region as ethnic markers, disregarding conceptual distinctions between these categories.

This heterogeneity in the operationalization of the race/ethnicity variable constitutes a significant source of methodological variability, potentially masking intra-group differences, compromising the precision of observed associations, and limiting comparability across studies, while also potentially contributing to the variations in reported prevalence. Due to the methodological approach adopted by the primary studies, the terms race and ethnicity were used interchangeably in this review to maintain coherence with the categories originally employed in the analyzed research.

The data showed that brown/black pregnant women and indigenous pregnant women have the highest prevalence of gestational toxoplasmosis [21]. Black women (black and brown) generally have less access to quality health services and are more vulnerable, which may result in fewer serological screenings for toxoplasmosis during pregnancy [31]. Brown-skinned pregnant women had higher seropositivity rates compared to white women [22, 32].

Women belonging to traditional peoples, such as indigenous women, face several challenges in accessing health services due to inadequate health infrastructure, a lack of trained health professionals, and the distance between specific health services and indigenous lands [33]. Thus, unfavorable socioeconomic conditions, environmental conditions, and social vulnerability contribute to the high prevalence of the disease among black and indigenous women.

Among the seven studies that performed multivariate adjustment, the association between race/ethnicity and toxoplasmosis remained statistically significant even after controlling for variables such as income, education, and sanitation, indicating that the association between race/ethnicity and gestational toxoplasmosis persisted after adjustment for the socioeconomic variables included in the analytical models. Although no direct association with racism or gender disparities was measured, the studies included in this review were not designed to evaluate the causal mechanisms underlying this association.

Differences in toxoplasmosis prevalence across studies may also reflect variations in socioeconomic conditions, environmental exposures, and cultural practices, including dietary habits and housing conditions. These factors are unevenly distributed across populations and may interact with broader social inequalities affecting racially and ethnically marginalized groups [34]. Such inequalities may be particularly relevant among women living in rural areas or Indigenous territories and among those with lower educational attainment [35].

Racism has been described as a system of oppression that sustains social hierarchy and limits accessibility to resources [36]. The literature corroborates this, evidencing that these mechanisms negatively impact health throughout the life course through social segregation and housing discrimination, which are associated with environmental exposure, maternal morbidity, and lack of access to prenatal care [37–39]. Furthermore, it contributes to inequities in access to services and in the quality of healthcare provided to social minorities [40].

Similarly, gender inequalities may interact with socioeconomic and ethno-racial factors in shaping contexts of vulnerability. Structural gendered racism contributes to prenatal care disparities and discriminatory obstetric care, disproportionately affecting Black women within health services [39, 41].

Thus, racism intersects with gender, race/ethnicity, and economic inequality, resulting in multifaceted disadvantages across all social systems. From the perspective of the social determinants of health, Black women or those from minority ethnic groups living in low-income conditions face diverse barriers to service access and quality of care, contributing to the prevalence differences observed among the population groups in the studies [42]. Recognizing this social determination is essential to highlight how hierarchies, power relations, and institutions sustain health inequities [43].

Among pregnant women of Asian origin, there was a widespread use of the term as a synonym for ethnicity. When using the term ‘Asian,' it is defined as individuals originating from any of the countries of Central, East, or South Asia [44]. Furthermore, studies that describe the categories ‘Middle East' and ‘Far East' as ethnicities contradict the conceptual definition of ethnicity, as they link a geographical area without considering the heterogeneity of the populations residing there.

The prevalence among Chinese women in the study by Liu et al. was 10.6% [45], a result that differed from the findings of the survey by Rostami et al. which reported a prevalence of 23.4% for Asian pregnant women, 35.1% for Middle Eastern pregnant women, and 11.8% for Far Eastern pregnant women [46]. This variation in prevalence between continents can be explained by the lack of a globally standardized protocol. It is clear that there is diversity in protocols among some countries: in Austria, there are mandatory national prenatal screening programs; while in countries such as the United Kingdom and the Netherlands, there are protocols focused on the adoption of educational measures; and in others, such as Denmark and Poland, the protocol is to perform screening in the neonatal period [47]. In some African countries, there are currently no specific national programs against toxoplasmosis in place [48].

However, it is noted that the majority of the included studies were conducted in the Americas and Asia, with scarce representation from African, European, and Oceanian countries. This geographical concentration limits the global generalizability of the findings, especially considering the distinct epidemiological, structural, and sociocultural dynamics that influence toxoplasmosis transmission and access to prenatal care across different regions. The absence of robust data from African contexts and certain European and Oceanian regions highlights significant gaps in the literature and reinforces the need for multicenter investigations and international collaborations to enhance population representation and methodological comparability across studies.

The variation in the prevalence of toxoplasmosis infection among pregnant women may be related to social, cultural, and environmental factors, including socioeconomic status, dietary habits, educational level, and limited knowledge about the routes of T. gondii transmission. Previous studies have identified risk factors such as consumption of raw salad or raw/undercooked meat, intake of untreated water, type of housing, and contact with cats [49–51].

Prevention plays a key role in avoiding congenital infection by T. gondii. In this context, preventive measures depend largely on women’s knowledge about toxoplasmosis, particularly regarding food hygiene, safe water consumption, and appropriate handling of environments where cats may defecate. These precautions should be reinforced during prenatal care [48, 52].

Another factor that may contribute to differences in prevalence is the age distribution of pregnant women. Some studies reported higher prevalence in age groups between 16 and 26 years, 31 years or older, and 32–45 years [19, 22, 32]. Overall, higher prevalence tends to occur among women of reproductive age, which may reflect greater exposure to risk factors associated with the infection [53].

Differences in prevalence may also be influenced by the diversity of diagnostic methods used across studies, as serological tests present different levels of sensitivity and specificity. These variations may affect the detection of infected pregnant women and contribute to differences in reported prevalence [54–56].

In addition, the IgG avidity test may influence the interpretation of serological results, as it helps distinguish between acute and chronic Toxoplasma gondii infection, contributing to a more accurate classification of infection timing [57, 58].

These findings highlight the importance of considering structural and social determinants when designing public health strategies aimed at reducing inequalities in maternal health and infectious disease prevention.

Additionally, the development of culturally sensitive educational interventions is recommended, taking into account the linguistic, religious, and sociocultural specificities of the served populations. Such strategies should include clear guidance on preventive measures—such as proper food hygiene, safe water consumption, and safe management of animal waste—integrated into prenatal care routines.

At the clinical-care level, we emphasize the need for a comprehensive and equitable approach, with explicit recognition of the intersectional vulnerabilities involving race/ethnicity, gender, and socioeconomic status. The qualification of health professionals to identify and address structural barriers in accessing serological screening is fundamental to mitigating the effects of institutional inequalities and promoting greater health justice.

### Study Limitations

Despite the relevance of the findings, this review presents limitations that must be considered when interpreting the results. High methodological heterogeneity was observed among the included studies regarding study designs, population characteristics, and analytical strategies, which precluded the performance of a meta-analysis and limits direct comparability between findings.

The geographic representation was concentrated in the Americas and Asia, with an underrepresentation of European and African countries, thus restricting the generalizability of the results to distinct global contexts.

Furthermore, inconsistencies were identified in the ethno-racial classifications used, involving different categorization criteria and measurement methods, which may introduce classification biases. Variability in the diagnostic methods employed may also have influenced the reported prevalence estimates.

It is also noteworthy that some studies did not perform adequate adjustment for potential confounding variables—such as socioeconomic conditions, access to healthcare services, and other social determinants—which may impact the magnitude of the observed associations between race/ethnicity and gestational toxoplasmosis.

Despite these limitations, this review was conducted with methodological rigor and contributes to highlighting gaps in the literature, reinforcing the need for future investigations with greater methodological standardization and an intersectional approach to social determinants.

### Conclusion

The studies included in this review suggest variations in the prevalence of gestational toxoplasmosis across different ethno-racial groups and geographic contexts. However, the interpretation of these differences must be conducted with caution, considering that a significant portion of the studies did not perform consistent adjustment for potential socioeconomic, environmental, and structural confounding factors.

The observed methodological heterogeneity—including variations in the operationalization of race/ethnicity, diagnostic methods, and analytical strategies—limits causal inferences regarding the independent role of race/ethnicity in the occurrence of toxoplasmosis during pregnancy. In this sense, the findings of this review should be understood as indicative of possible inequalities in the distribution of the infection, rather than as conclusive evidence of an independent association.

Above all, this review highlights significant gaps in the literature concerning the conceptual and methodological standardization of the race/ethnicity variable and the adequate control of social determinants of health. Such limitations hinder the identification of explanatory mechanisms and the adequate interpretation of the observed differences across populations.

Future investigations should prioritize more robust analytical designs, featuring systematic adjustment for social determinants, standardization in race/ethnicity classification, and transparent reporting of the operationalization of these variables. Longitudinal studies and stratified analyses with control for confounders may contribute more consistently to understanding the relative role of race/ethnicity in the epidemiology of gestational toxoplasmosis.
